# Clinical value of luciferase-based bioluminescence assay in diagnosis of Alport syndrome

**DOI:** 10.3389/fped.2026.1706611

**Published:** 2026-03-12

**Authors:** Yue Cai, Ying-qi Lin, Xin-yu Kuang, Lei Sun, Meng-ying Li, Yu-jie Hu, Wen-yan Huang

**Affiliations:** Department of Nephrology, Rheumatology and Immunology, Shanghai Children’s Hospital, School of Medicine, Shanghai Jiao Tong University, Shanghai, China

**Keywords:** Alport syndrome, diagnosis, genetic variation, luciferases, pediatrics

## Abstract

**Objectives:**

Alport syndrome (AS) is an inherited kidney disorder caused by pathogenic variants in *COL4A3*, *COL4A4*, or *COL4A5*. In this study, we aim to apply a split-luciferase bioluminescence assay to functionally assess *COL4A3*, *COL4A4*, or *COL4A5* variants of uncertain significance (VUS) identified in pediatric patients with Alport syndrome, and to explore its value in supporting variant interpretation and diagnostic evaluation.

**Methods:**

A retrospective analysis included 31 children who met established clinical and pathological diagnostic criteria for Alport syndrome, but in whom genetic testing identified VUS in *COL4A3*, *COL4A4*, or *COL4A5*. Genomic DNA was analyzed using next-generation sequencing (NGS) to identify variant loci. Recombinant plasmids corresponding to the identified variants were constructed and transfected into HEK293T cells. The split-luciferase bioluminescence assay (NanoBiT® system) was employed to measure luminescence signals in living cells. Luminescence intensity was compared between the VUS-associated plasmids and the wild-type (WT) plasmid, and the sensitivity and specificity of this technique were evaluated.

**Results:**

The luminescence intensity of all 31 plasmids carrying VUS identified in children with Alport syndrome was reduced by more than 50% compared with the WT plasmid group (*P* < 0.01, *n* = 3). When the luminescence intensity was below 397.8, the sensitivity for distinguishing VUS from WT was 96.77%, and the specificity was 100%.

**Conclusions:**

The split-luciferase bioluminescence assay was successfully applied as an *in vitro* functional approach to assess *COL4A3*, *COL4A4*, or *COL4A5* VUS, and may reliably assist in the diagnostic evaluation of pediatric patients with suspected Alport syndrome whose genetic testing reveals VUS, particularly in cases where pathological assessment is unavailable or not feasible.

## 1 Introduction

Alport syndrome (AS) is an inherited kidney disorder caused by pathogenic variants in *COL4A3, COL4A4*, or *COL4A5*, which encode the α-chains of type IV collagen. Clinical manifestations mainly include hematuria, proteinuria, and progressive renal failure, and some pediatric patients also present with hearing and ocular abnormalities (1, 2). The diagnosis of AS primarily relies on persistent glomerular hematuria, hematuria with proteinuria, family history, characteristic basement membrane alterations on electron microscopy, abnormal α-chain expression, and genetic testing (3).

However, in a substantial proportion of pediatric cases, genetic testing identifies variants of uncertain significance (VUS), which poses challenges for definitive diagnosis. When pathogenic or likely pathogenic variants are identified, genetic testing provides strong diagnostic evidence (4). In contrast, when genetic testing identifies only VUS, functional validation becomes essential for clarifying variant pathogenicity and supporting the final diagnosis. Current clinical functional validation methods involve invasive procedures, such as kidney biopsy or skin biopsy, which may be declined by families or considered unsuitable in certain clinical situations (5). At present, there is no precise and non-invasive functional validation method that can reliably assist in the diagnostic evaluation of pediatric patients with suspected AS whose genetic testing reveals VUS.

Previous studies have demonstrated the feasibility of using a split-luciferase bioluminescence assay to evaluate the trimer polymerization capacity of clinically relevant type IV collagen α5 variants (6). Based on this, this study applies this assay to functionally evaluate *COL4A3*, *COL4A4*, and *COL4A5* VUS identified in pediatric patients diagnosed with AS according to established clinical and pathological criteria in order to support variant interpretation and diagnostic evaluation, particularly when pathological evaluation is unavailable.

## 2 Materials and methods

### 2.1 Patients and data collection

A retrospective analysis was conducted on 31 pediatric patients with Alport syndrome who presented to our department between May 2014 and August 2022. The inclusion criteria were as follows:

(1) Meeting the diagnostic criteria of the Chinese Expert Consensus on the Diagnosis and Treatment of Alport Syndrome (2023 Edition), namely persistent glomerular hematuria with or without proteinuria, together with at least one of the following findings:
  (a) characteristic ultrastructural alterations of the glomerular basement membrane (GBM) on electron microscopy, including splitting and lamellation of the lamina densa, irregular thinning and thickening, or a basket-weave pattern;
  (b) abnormal immunofluorescence staining of type IV collagen α3, α4, or α5 chains in the renal basement membrane, or abnormal immunofluorescence staining of the type IV collagen α5 chain in extrarenal basement membranes (EBM);
  (c) identification of a pathogenic or likely pathogenic variant in *COL4A3*, *COL4A4*, or *COL4A5* according to ACMG criteria;

With the exception that in the present cohort, genetic testing did not identify any pathogenic or likely pathogenic variants in *COL4A3*, *COL4A4*, or *COL4A5*.

(2) availability of functional validation by skin or kidney biopsy demonstrating abnormal α-chain expression;
(3) Genetic testing revealed VUS in *COL4A3*, *COL4A4*, or *COL4A5*.

Clinical and family data were collected, including patient sex, clinical manifestations, age and mode of onset, diagnostic and therapeutic course, time and method of diagnosis, family genetic history, urinalysis, kidney function assessment, fundus examination, hearing evaluation, and pathological kidney biopsy findings (light microscopy, immunofluorescence, and electron microscopy). Variant information was verified using OMIM, ClinVar, and HGMD databases, and pathogenicity was assessed per ACMG guidelines.

### 2.2 Next-generation sequencing (NGS) and variant analysis

Genomic DNA was extracted from peripheral blood leukocytes using the GentraPuregene Kit (Qiagen, Germany) according to the manufacturer's protocol. Targeted next-generation sequencing (NGS) of *COL4A3*, *COL4A4*, and *COL4A5* was performed on the Illumina platform following standard library preparation and hybrid-capture procedures. Sequencing data were aligned to the human reference genome (GRCh37/hg19), and single-nucleotide variants and small indels were called using a standard bioinformatics pipeline. Variants were annotated and interpreted according to ACMG guidelines, with in silico prediction of protein function performed using SIFT and PolyPhen-2. Candidate variants were confirmed by Sanger sequencing of PCR-amplified fragments covering the variant sites.

### 2.3 Wild-Type *COL4A3*/*A4*/*A5* plasmids

Based on the coding DNA sequences (CDS) of *COL4A3* (NM_000091.5), *COL4A4* (NM_000092.5), and *COL4A5* (NM_033380.3) obtained from the NCBI database (https://www.ncbi.nlm.nih.gov/), primers were designed and PCR amplification, restriction digestion and ligation, positive clone screening, and Sanger sequencing were performed to generate wild-type expression plasmids for subsequent experiments.

For the split-luciferase assay, fusion plasmids were generated by inserting the *COL4A3* coding region into the pBiT2.1-C [TK/SmBiT] vector and the *COL4A5* coding region into the pBiT1.1-C [TK/LgBiT] vector. The small (SmBiT) and large (LgBiT) luciferase fragments were fused to the C-termini of the *COL4A3* and *COL4A5* coding regions, respectively. Meanwhile, * COL4A4* was subcloned into the pcDNA3.1-3Flag expression vector.

### 2.4 Variant *COL4A3/A4/A5* plasmids

Site-directed mutagenesis primers were designed according to the variant information identified in clinical patients, using SnapGene software (GSL Biotech, USA). Site-directed mutagenesis was performed on the wild-type *COL4A3*, *COL4A4*, and *COL4A5* plasmids using the Mut Express® II Fast Mutagenesis Kit V2 (Vazyme, Cat. # C214, China). The resulting plasmids were purified and verified by Sanger sequencing. Endotoxin-free plasmid minipreps were performed, and the recombinant plasmids were stored at −20 °C.

### 2.5 Cell transfection

HEK293 T cells (ATCC, Cat. # CRL-3216, USA) were passaged to passages 3–4 and seeded into 96-well plates when cells were healthy and sub-confluent. Cells were divided into six groups:

(1) blank control (cells without plasmid transfection),
(2) positive control (PRKACA–SmBiT/PRKAR2A–LgBiT constitutive interacting pair supplied by the NanoBiT® PPI system),
(3) negative control (NanoBiT® Negative Control Vector, HaloTag–SmBiT),
(4) wild-type (WT) plasmid,
(5) pathogenic variant plasmid,
(6) experimental variant (VUS) plasmids.

For each variant and control sample, three technical replicate wells were prepared within the same assay run. Plasmid DNA for each group, except the blank control, was transiently transfected using Lipo8000™ Transfection Reagent (Beyotime, Cat. # C0533, China), following the manufacturer's instructions. After 48 h of culture post-transfection, transfection efficiency was assessed under a fluorescence microscope.

### 2.6 Split-luciferase bioluminescence assay

Luminescence detection was performed using the NanoBiT® PPI MCS Starter System (Promega, Cat. # N2014, USA). Nano-Glo® Live Cell Reagent (25 µL per well) was added to 96-well white plates at room temperature, and luminescence was measured using a GloMax® Navigator microplate luminometer (Promega, USA).

### 2.7 Statistical analysis

Statistical analyses were performed using GraphPad Prism (GraphPad Software, USA). Data are presented as mean ± SD. ANOVA was applied for comparisons among multiple groups, whereas Student's t-test was used for comparisons between each VUS-associated plasmid and the WT plasmid. A *P*-value < 0.05 was considered statistically significant.

To evaluate the diagnostic performance of the split-luciferase bioluminescence assay for VUS, ROC curve analysis was performed. For each of the 31 VUS variants, the mean luminescence value from three technical replicate wells was calculated and used as a single data point representing the VUS-associated plasmid, while the WT plasmid served as the reference control. The optimal luminescence cutoff value was determined using the Youden index. Sensitivity and specificity were calculated relative to the WT control.

## 3 Results

### 3.1 Clinical data information

From May 2014 to August 2022, 31 pediatric patients who met the inclusion criteria were evaluated in our department, comprising 15 boys and 16 girls. The mean age at disease onset was 5.7 ± 3.5 years, and the mean follow-up duration was 5.5 ± 2.2 years (Table 1). Among the identified variants, 23 (74.2%) were located in *COL4A5*, 7 (22.6%) in *COL4A3*, and 1 (3.2%) in *COL4A4* (Table 2).

**Table 1:** Clinical information of variants in enrolled children with Alport syndrome.

Patient ID	Sex	Onset type	Age at onset (y)	Follow-up (y)	Kidney biopsy		Skin biopsy
					Light microscopy	Electron microscopy	
A501	F	Microscopic hematuria	3	5	α3, α5 focally positive	/	/
A502	F	Microscopic hematuria	9	4	/	GBM lamination and splitting	/
A503	F	Microscopic hematuria	3	4	/	Mesangial matrix expansion in glomerular capillary loops	/
A504	F	Hematuria with proteinuria	7	6	α5 negative	/	/
A505	M	Hematuria	3	4	α5 negative	/	/
A506	M	Microscopic hematuria	2	8	α3 reduced, partially absent; α5 negative	/	/
A507	F	Gross hematuria	17	4	α3, α5 negative	/	/
A508	M	Microscopic hematuria	6	9	α3, α5 focally positive	/	/
A509	M	Hematuria	6	3	α5 negative	/	/
A510	F	Microscopic hematuria	8	8	α5 negative	/	/
A511	M	Hematuria	14	4	α3, α5 negative	/	/
A512	F	Hematuria with proteinuria	4	1	/	/	α5 completely absent
A513	M	Gross hematuria	5	8	α5 negative	/	/
A514	F	Hematuria with proteinuria	3	4	α3, α5 focally positive	/	/
A515	F	Microscopic hematuria	3	7	α3, α5 focally positive	/	/
A516	M	Gross hematuria	2	8	α3, α5 negative	Mesangial proliferation	/
A517	M	Microscopic hematuria	6	4	α3 reduced, partially absent α5 negative	/	/
A518	M	Proteinuria	8	1	/	FSGS	α5 focally lost
A519	M	Microscopic hematuria	6	5	α3, α5 negative	/	/
A520	F	Hematuria with proteinuria	3	4	α3, α5 focally positive	/	/
A521	F	Hematuria with proteinuria	3	7	α3 focally positive	mild mesangial proliferation	/
A522	F	Microscopic hematuria	8	5	α3 focally positive	mild mesangial proliferation	/
A523	M	Microscopic hematuria	3	8	α3, α5 negative	/	/
A301	F	Microscopic hematuria	12	8	α3, α5 focally positive	/	/
A302	M	Gross hematuria	7	8	α3, α5 focally positive	mild glomerular lesions	/
A303	F	Microscopic hematuria	4	7	α3, α5 reduced	mild glomerular lesions	/
A304	M	Hematuria with proteinuria	8	5	α3, α5 negative	focal segmental GBM lamination	/
A305	F	Hematuria with proteinuria	2	7	α3 focally positive, α5 negative	/	/
A306	M	Hematuria with proteinuria	4	3	α3, α5 negative	/	/
A307	M	Microscopic hematuria	3	2	α5 completely absent; α3 focally positive	GBM lamination and splitting	α5 completely absent
A401	F	Microscopic hematuria	6	8	α3, α5 focally positive	/	/

F, female; M, male.

**Table 2:** Gene variants and pedigree verification of enrolled children with Alport syndrome.

Patient ID	Gene	Exon Location	Nucleotide Change	Amino Acid Change	Mutation origin
A501	*COL4A5*	EX04	c.262C > T	p.Pro88Ser	Father
A502	*COL4A5*	EX06	c.349G > A	p.Gly117Arg	Mother
A503	*COL4A5*	EX06	c.358G > A	p.Gly120Ser	Mother
A504	*COL4A5*	EX10	c.548G > A	p.Gly183Asp	*De novo*
A505	*COL4A5*	EX10	c.592G > A	p.Gly198Arg	Mother
A506	*COL4A5*	EX10	c.601G > C	p.Gly201Arg	Mother
A507	*COL4A5*	EX13	c.688G > A	p.Gly230Ser	Mother
A508	*COL4A5*	EX17	c.938G > T	p.Gly313Val	Mother
A509	*COL4A5*	EX20	c.G1225A	p.G409S	Mother
A510	*COL4A5*	EX26	c.1967G > A	p.Gly656Asp	Mother
A511	*COL4A5*	EX28	c.2174G > A	p.Gly725Glu	Mother
A512	*COL4A5*	EX30	c.2441G > A	p.Gly814Glu	Father
A513	*COL4A5*	EX31	c.2519G > A	p.Gly840Glu	Mother
A514	*COL4A5*	EX32	c.2678G > A	p.Gly893Asp	*De novo*
A515	*COL4A5*	EX32	c.2749G > A	p.Gly917Ser	Mother
A516	*COL4A5*	EX33	c.2800_2805delCCTGGC	p.934_935delProGly	*De novo*
A517	*COL4A5*	EX33	c.2822G > A	p.Gly941Asp	Mother
A518	*COL4A5*	EX36	c.3155A > C	p.Gln1052Pro	Mother
A519	*COL4A5*	EX37	c.3248G > A	p.Gly1083Asp	Mother
A520	*COL4A5*	EX43	c.3940C > T	p.Pro1314Ser	Mother
A521	*COL4A5*	EX47	c.4316G > T	p.Gly1439Val	*De novo*
A522	*COL4A5*	EX49	c.4408A > G	p.lle1470Val	Mother
A523	*COL4A5*	EX50	c.4975A > G	p.Ser1659Gly	Mother
A301	*COL4A3*	EX16	c.898G > A	p.Gly300Arg	Mother
A302	*COL4A3*	EX20	c.1150G > A	p.Gly384Arg	Father
A303	*COL4A3*	EX32	c.2555C > T	p.Pro852Leu	*De novo*
A304	*COL4A3*	EX34	c.2864G > A	p.Gly955Glu	Mother
A305	*COL4A3*	EX47	c.4199G > A	p.Gly1400Glu	Father
A306	*COL4A3*	EX50	c.4664C > T	p.Alal555Val	*De novo*
A307	*COL4A3*	EX50	c.4669G > C	p.Ala1557Pro	Mother
A401	*COL4A4*	EX21	c.1396G > A	p.Gly466Arg	Mother

In addition, one male patient carrying a pathogenic *COL4A5* variant was included as a reference pathogenic control. This patient presented with gross hematuria at 5 months of age, subsequently developed sensorineural hearing loss, and progressed to end-stage renal disease at 16 years of age. Skin biopsy immunofluorescence demonstrated preserved type IV collagen α1 expression in the basement membrane with complete absence of α5 expression. Genetic testing identified a pathogenic frameshift variant in *COL4A5* (c.2440delG, p.Gly814fs).

### 3.2 Screening of optimal fusion orientation for the split-luciferase assay

**Figure 1:**
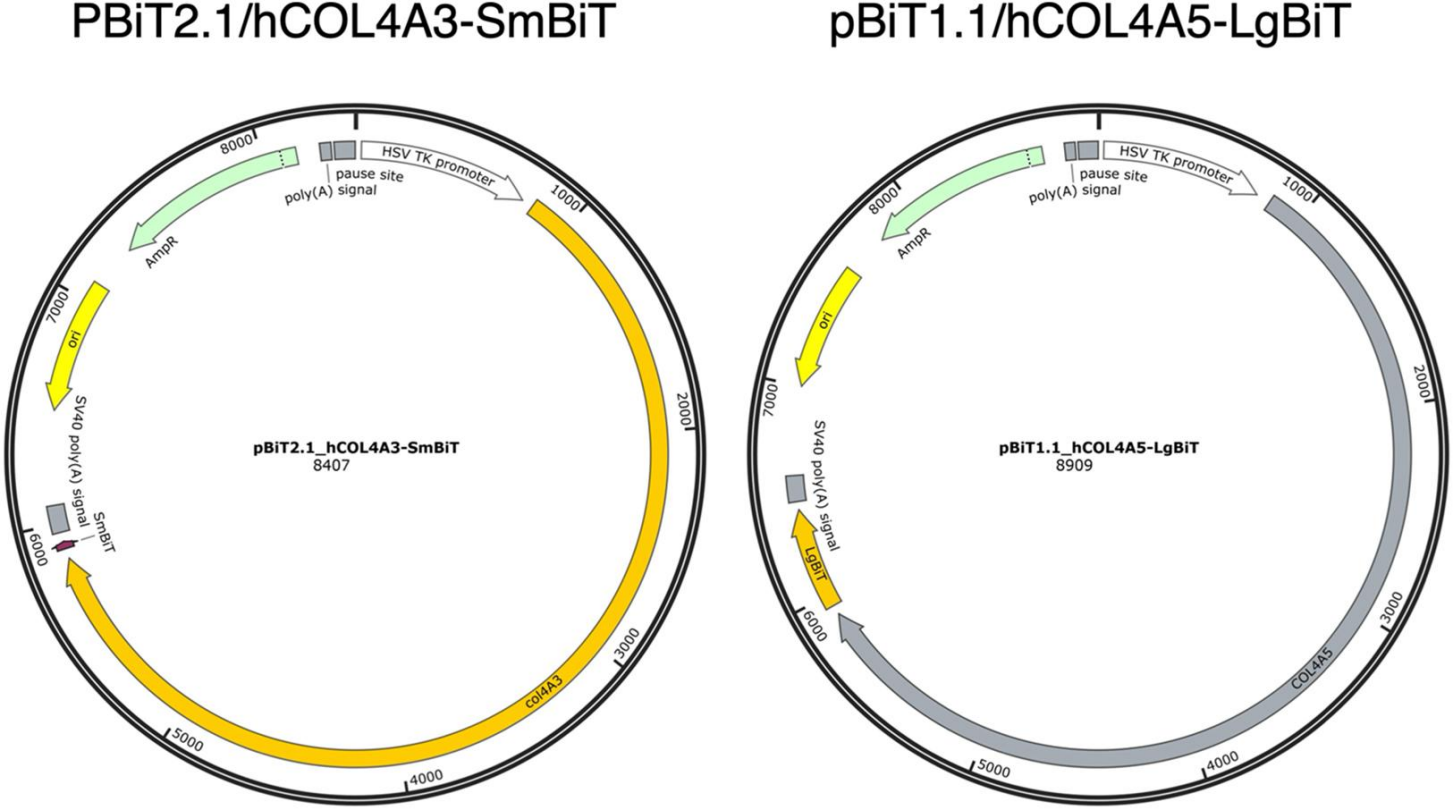
Structure of plasmid *COL4A3*-smBiT and *COL4A5*-lgBiT.

*COL4A3* was fused with SmBiT and *COL4A5* with LgBiT at their C-termini, respectively, to determine the optimal configuration for detecting α3α4α5(IV) trimers. In the presence of *COL4A4*, the *COL4A3*-SmBiT/*COL4A5*-LgBiT pair exhibited the highest luminescence intensity. The results are illustrated in (Figure 1).

### 3.3 Verification of recombinant plasmids carrying *COL4A3*, *COL4A4*, and *COL4A5* variants

**Figure 2:**
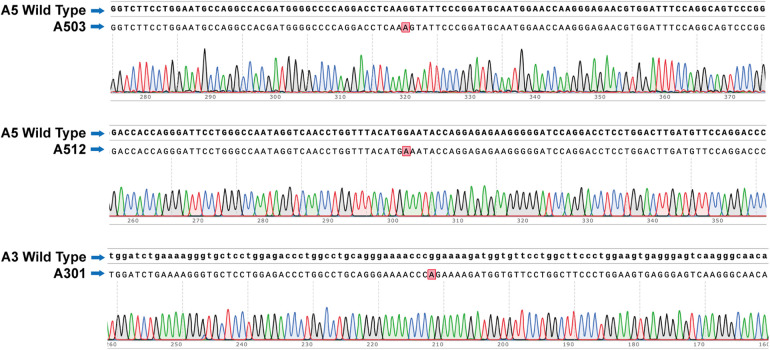
Sequencing results of recombinant plasmids carrying *COL4A3*, *COL4A4*, and *COL4A5* variants. Footnote: the variant plasmids A501, A512, and A301 in the figure above are taken as examples

Restriction enzyme digestion confirmed the successful construction of recombinant plasmids carrying *COL4A3*, *COL4A4*, and *COL4A5* variants. Sanger sequencing verified that no additional nucleotide or amino acid substitutions occurred outside the intended variant sites (Figure 2).

### 3.4 Luminescence expression levels of *COL4A3*, *COL4A4*, and *COL4A5* variant plasmids

Following cell transfection, luminescence signals from live cells were measured using the split-luciferase bioluminescence assay. The luminescence intensities for each plasmid group were summarized in Table 3.

**Table 3:** Luminescence intensities of WT, VUS-associated, and pathogenic variant plasmids.

ID	Gene	Luminescence intensity (mean ± SD)	Adjusted *P* Value
WT		1,382.33 ± 97.18	
P501	*COL4A5*	56.00 ± 8.19	<0.0001
A501	*COL4A5*	102.67 ± 11.84	<0.0001
A502	*COL4A5*	119.67 ± 4.03	<0.0001
A503	*COL4A5*	176.00 ± 46.88	<0.0001
A504	*COL4A5*	115.33 ± 17.52	<0.0001
A505	*COL4A5*	59.67 ± 16.36	<0.0001
A506	*COL4A5*	282.67 ± 69.81	<0.0001
A507	*COL4A5*	172.33 ± 59.02	<0.0001
A508	*COL4A5*	156.00 ± 30.69	<0.0001
A509	*COL4A5*	288.67 ± 74.07	<0.0001
A510	*COL4A5*	72.67 ± 7.72	<0.0001
A511	*COL4A5*	82.33 ± 27.52	<0.0001
A512	*COL4A5*	71.67 ± 37.04	<0.0001
A513	*COL4A5*	323.67 ± 17.44	<0.0001
A514	*COL4A5*	66.67 ± 16.66	<0.0001
A515	*COL4A5*	89.33 ± 21.93	<0.0001
A516	*COL4A5*	472.00 ± 119.73	<0.0001
A517	*COL4A5*	123.00 ± 23.04	<0.0001
A518	*COL4A5*	97.33 ± 21.82	<0.0001
A519	*COL4A5*	81.00 ± 15.94	<0.0001
A520	*COL4A5*	85.33 ± 3.86	<0.0001
A521	*COL4A5*	88.00 ± 7.12	<0.0001
A522	*COL4A5*	86.33 ± 3.09	<0.0001
A523	*COL4A5*	98.33 ± 27.21	<0.0001
A301	*COL4A3*	88.67 ± 22.88	<0.0001
A302	*COL4A3*	70.67 ± 10.66	<0.0001
A303	*COL4A3*	70.33 ± 11.90	<0.0001
A304	*COL4A3*	98.00 ± 42.62	<0.0001
A305	*COL4A3*	78.67 ± 14.38	<0.0001
A306	*COL4A3*	92.00 ± 5.35	<0.0001
A307	*COL4A3*	94.00 ± 9.20	<0.0001
A401	*COL4A4*	99.67 ± 13.30	<0.0001

Variants labeled with the prefix “A” represent plasmids carrying VUS, variants labeled with the prefix “P” represent plasmids carrying pathogenic variants.

**Figure 3:**
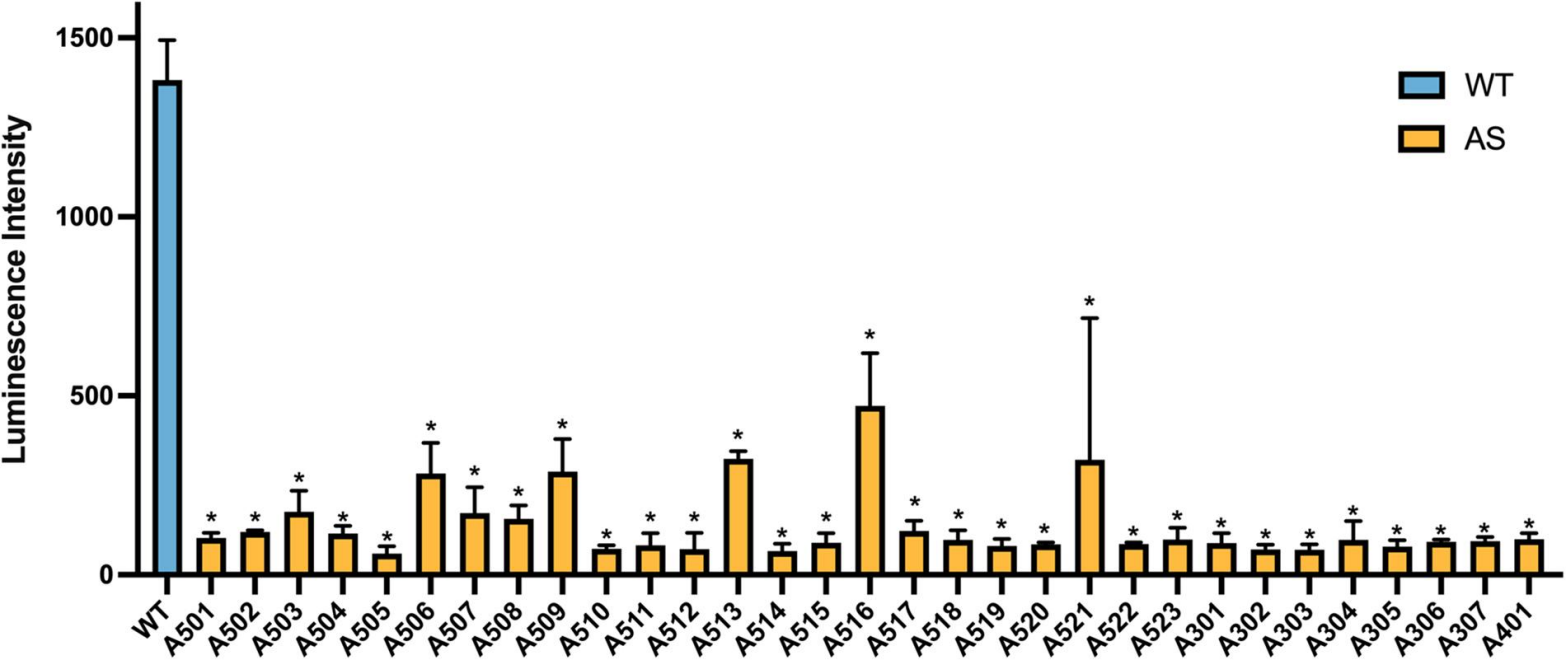
The luminescence intensity of VUS-associated plasmids and the WT plasmid (*P < 0.01, *n* = 3). Footnote: asterisks above VUS plasmids indicate statistical significance compared to the WT plasmid.

Comparison of luminescence intensities between the above variant recombinant plasmids of *COL4A3*, *COL4A4*, and *COL4A5* and the WT plasmid demonstrated that all 31 VUS-associated plasmids exhibited a reduction of more than 50% in luminescence relative to WT. Statistical analysis revealed significant differences between each variant plasmid and the WT plasmid (*P* < 0.01). Further ROC analysis indicated that when the luminescence intensity was below 397.8, the assay distinguished VUS-associated plasmids from the WT plasmid with a sensitivity of 96.77% and a specificity of 100% (Figure 3).

## 4 Discussion

AS typically presents in childhood with persistent hematuria, followed by proteinuria, progressive decline in kidney function, and eventual progression to renal failure. In addition to renal involvement, children with Alport syndrome often develop extra-kidney manifestations, including sensorineural hearing loss during childhood and adolescence, while ocular abnormalities generally appear in adulthood (7–9). Currently, no disease-specific therapy is available for AS. Clinically, blood pressure control and reduction of proteinuria are the main strategies to delay the progression of chronic kidney disease (10, 11).The median age at renal failure in males with X-linked AS (XLAS) is approximately 25 years, and about 90% of patients require kidney replacement therapy before the age of 40 due to progression to renal failure. In Europe, AS accounts for 1%–2% of kidney transplant recipients, making it one of the leading causes of renal failure (12, 13).

Both clinical trials and animal studies have shown that early pharmacologic intervention, initiated before the onset of proteinuria, can delay kidney function decline and improve long-term prognosis (14). Gross et al. reported that early administration of ACE inhibitors in pediatric patients with AS effectively delays kidney function deterioration (15). Therefore, early diagnosis and prompt intervention are essential to slow disease progression in pediatric AS (16). With the rapid advancement of molecular diagnostic techniques, molecular genetic testing has become an effective diagnostic approach for AS. Genetic testing methods have evolved from single-gene Sanger sequencing to widely used next-generation sequencing (NGS), significantly improving the molecular diagnostic accuracy for AS. To date, based on the ClinVar database (https://www.ncbi.nlm.nih.gov/clinvar), the *COL4A3* and *COL4A4*, located on chromosome 2, have over 430 and 325 pathogenic and likely pathogenic variants associated with AS, respectively, most commonly causing autosomal recessive inheritance and rarely autosomal dominant inheritance. In addition to these, approximately 314 and 277 variants in *COL4A3* and *COL4A4* are currently classified as VUS. *COL4A5*, located on the X chromosome, has over 768 reported pathogenic and likely pathogenic variants associated with AS, together with 209 variants classified as VUS. Despite these advances, several challenges remain in the molecular diagnosis of AS, including technical limitations in sequencing, bioinformatics interpretation, and particularly the functional validation of VUS. Functional validation of VUS is currently the major barrier to clinical translation of genetic results (17).

In this study, we examined 31 pediatric patients who met established clinical and pathological diagnostic criteria for AS. Genetic testing in these patients did not identify pathogenic or likely pathogenic variants in *COL4A3*, *COL4A4*, or *COL4A5*, but instead revealed VUS. These VUS consisted predominantly of missense substitutions (*n* = 30), together with one deletion variant. The luminescence intensities of their variant *COL4* gene plasmids were measured *in vitro* using the split-luciferase bioluminescence assay. All variant plasmids demonstrated significantly reduced luminescence compared with the wild type. When luminescence intensity was < 397.8, the sensitivity and specificity for distinguishing VUS-associated plasmids from WT were 96.77% and 100%, respectively. These findings demonstrate that the split-luciferase assay can distinguish constructs carrying VUS from the WT at the functional level, providing a quantitative readout of variant-related effects in this experimental system.

Alport syndrome is clinically heterogeneous, with manifestations ranging from isolated hematuria to hematuria accompanied by proteinuria and progressive renal impairment (18). In clinical practice, kidney or skin biopsy is not always performed because of its invasive nature, parental consent considerations, or limited clinical indications. Consequently, when genetic testing identifies only VUS, establishing a definitive diagnosis based solely on clinical features and sequencing results can be challenging. In this context, the reduced luminescence observed for VUS-associated plasmids in the split-luciferase assay provides supportive functional evidence that may assist diagnostic evaluation in selected cases, particularly when biopsy is unavailable, deferred, or not feasible. However, this study has limitations, including a relatively small sample size. Further research with larger cohorts is necessary to validate the feasibility and reliability of the split-luciferase assay for routine AS diagnosis. Moreover, although kidney or skin biopsy with electron microscopy is considered the important diagnostic component for AS diagnosis, the rate of typical pathological changes in pediatric patients is relatively low, and some cases may still be missed. The split-luciferase bioluminescence assay offers a promising complementary method that may reduce the risk of missed diagnoses in AS.

## 5 Conclusions

We successfully utilized the split-luciferase bioluminescence assay to functionally validate and assess *COL4A3*, *COL4A4*, and *COL4A5* variants of uncertain significance in children with Alport syndrome. This *in vitro* assay provides supportive functional evidence for variant interpretation and may assist the final diagnosis, particularly in clinical situations where biopsy is unavailable or not feasible.

## Data Availability

The original contributions presented in the study are publicly available. This data can be found here: National Center for Biotechnology Information (NCBI) ClinVar, https://www.ncbi.nlm.nih.gov/clinvar, accession numbers SCV007496364 - SCV007496394.
